# Stem-like CD4 T cells in tumors: an emerging framework for antitumor immunity

**DOI:** 10.3389/fimmu.2026.1853163

**Published:** 2026-08-27

**Authors:** Jiaxiang Xu, Yunchao Yang, Jun Zhang, Yue Zhang

**Affiliations:** 1 Affiliated Hospital of Shandong University of Traditional Chinese Medicine, Jinan, China; 2 Shandong College of Traditional Chinese Medicine, Yantai, Shandong, China; 3 Shandong University of Traditional Chinese Medicine, Jinan, China

**Keywords:** cancer immunotherapy, immune checkpoint blockade, stem-like CD4 T cells, T cell differentiation, T cell stemness, TCF1, tumor immunity, tumor microenvironment

## Abstract

Recent studies have identified antigen-experienced TCF1^+^PD-1^+^ lineage-negative (lin^-^) CD4 T cells in several tumor types. Strictly, stemness requires durable self-renewal and multipotent output demonstrated at the single-cell level; these criteria have not yet been established for an individual tumor-associated CD4 T cell. Operationally, this review uses “stem-like CD4 T cells” for TCF1^+^PD-1^+^ lin^-^ populations that lack canonical terminal TH1, TH2, TH17, and Treg commitment programs and show population-level persistence and multilineage output. Pending single-cell lineage tracing, these populations are best regarded as candidate progenitor-like states. Within the tumor microenvironment, their differentiation trajectory may influence antitumor immunity. Treg-mediated suppression, TGF-β signaling, metabolic stress, and limited IL-12 can restrain effector differentiation, whereas release of these constraints can permit TH1 output, CD8-supporting activity in tumor-draining lymph nodes, and direct MHC class II–restricted antitumor effects in selected models. Direct tumor evidence remains concentrated in a limited set of studies, while infection, autoimmunity, transplantation, and CD8 research provide contextual or cross-lineage support. This review therefore presents stem-like CD4 biology as an emerging framework and distinguishes established observations from working models.

## 1 Introduction

CD4 T cells are central to adaptive immunity, coordinating immune responses through differentiation into specialized subsets. Following the seminal identification of TH1 and TH2 cells by Mosmann and Coffman in the 1980s, the CD4 T cell repertoire has expanded to include TH17, follicular helper T (Tfh), regulatory T (Treg), Th9, Th22, and cytotoxic CD4 T cell subsets. These populations are defined by distinct transcriptional programs, cytokine profiles, and effector functions (1–6). This subset-based framework has substantially refined our understanding of how CD4 T cells tailor immune responses to diverse pathogenic and immunological contexts.

In tumors, CD4 T cells have historically been overshadowed by CD8 cytotoxic T lymphocytes, which mediate direct tumor cell killing via recognition of MHC class I–presented antigens (7, 8). However, accumulating evidence demonstrates that CD4 T cells are essential for effective antitumor immunity. Expansion of conventional CD4 T cells following checkpoint blockade, as well as their enrichment within tertiary lymphoid structures, is associated with improved clinical outcomes (9, 10). TH1 polarization enhances antigen presentation, activates macrophages and NK cells, and supports the priming and effector functions of CD8 T cells (11–13). In contrast, regulatory T (Treg) cells within the tumor microenvironment suppress antitumor responses and are frequently linked to disease progression (14, 15). A key unresolved question is what governs the balance between protective and suppressive CD4 T cell fates in tumors.

Although the conventional subset-based framework has been instrumental, it does not fully account for several observations in tumor immunology. First, tumor-infiltrating CD4 T cells rarely adopt canonical TH1 effector states and, unlike CD8 T cells, do not display a typical exhaustion phenotype (16, 17). Second, a substantial fraction expresses activation markers yet lacks lineage-defining transcription factors, consistent with an “uncommitted” state not captured by classical classifications (18, 19). Third, the mechanisms sustaining long-term CD4 T cell responses under chronic antigen exposure in tumors remain poorly defined. Together, these findings indicate the need for a framework that extends beyond traditional effector subset paradigms.

Strictly, stemness requires durable self-renewal together with multipotent output demonstrated at the single-cell level. These criteria have not yet been fully established for an individual tumor-associated CD4 T cell. Operationally, this review uses the term stem-like CD4 T cells for antigen-experienced TCF1^+^PD-1^+^ lin^-^ populations that show proliferative persistence and population-level evidence of multilineage differentiation (16, 18, 20, 21). Here, lin^-^ denotes the absence of canonical terminal lineage-commitment programs—operationally, lack of TBET, GATA3, RORγt, and FOXP3—rather than the absence of all lineage-associated factors. BCL6 is not included in this exclusion set because its expression is context dependent and can overlap with TCF1 in progenitor-like CD4 T cells (22). Until single-cell lineage tracing demonstrates both durable self-renewal and multipotent output in the same cell, these populations should be regarded as candidate progenitor-like states rather than definitively proven multipotent stem cells.

This review provides a comprehensive analysis of stem-like CD4 T cells in tumor immunology. We first outline the evolution of the T cell stemness concept from CD8 to CD4 lineages, and then summarize the molecular and metabolic features that define this population. We next examine how the tumor microenvironment shapes their differentiation, with emphasis on the Treg-mediated “restraint” versus “activation” paradigm and the CD4–CD8 cooperative axis underlying effective antitumor responses. Finally, we discuss therapeutic implications and highlight key outstanding questions for future investigation.

## 2 Evolution of T cell stemness

### 2.1 Defining stemness: insights from somatic stem cells

The concept of stemness in immunology derives from extensive studies of adult somatic stem cells in tissues such as the intestine, bone marrow, hair follicles, and skeletal muscle (23–26). These cells are defined by key properties: (i) self-renewal, enabling maintenance of the stem cell pool; (ii) differentiation capacity, generating specialized progeny to sustain tissue homeostasis; (iii) long-term persistence with relative quiescence; and (iv) dependence on niche-derived signals, including Wnt and Notch pathways, that preserve stem cell identity (27, 28). Stem cells may be multipotent or unipotent, but both exhibit developmental plasticity that distinguishes them from terminally differentiated cells.

Importantly, stemness is now viewed as a continuum rather than a binary state (29, 30). Single-cell RNA sequencing has demonstrated that stem cell compartments are heterogeneous and transitionally linked to their differentiated progeny (31). In hematopoiesis, long-term reconstituting hematopoietic stem cells (LT-HSCs) represent the most stem-like population, whereas multipotent progenitors (MPPs) retain partial stem-like features but exhibit increased differentiation bias (32, 33). This stem cell–progenitor continuum provides a useful framework for interpreting “stem-like” states in T cell populations.

### 2.2 Establishment of the stemness paradigm in CD8 T cells

The application of stem cell concepts to T cells began with the identification of T memory stem cells (TSCM) in graft-versus-host disease models (34). Derived from naïve T cells after antigen exposure, TSCM cells retain naïve-like phenotypes (CD44^lo CD62L^hi) while expressing activation markers such as CD95, CD122, and CXCR3 (35, 36). Functionally, they exhibit robust self-renewal and can reconstitute the full spectrum of memory T cell subsets, positioning them at the apex of the memory T cell hierarchy.

A major advance came in 2016, when three seminal studies identified a subset of virus-specific CD8 T cells expressing TCF1 during chronic LCMV infection that function as progenitors (37–39). These TCF1^+^ cells are enriched in lymphoid tissues, retain self-renewal and differentiation capacity, and constitute the primary responders to PD-1 blockade (38, 39). Unlike terminally exhausted CD8 T cells, which have limited proliferative potential, TCF1^+^ progenitors sustain responses through continuous generation of effector progeny. This work reframed T cell exhaustion as a differentiation continuum, progressing from stem-like progenitors to increasingly dysfunctional states, rather than a binary loss of function.

In tumor settings, stem-like CD8 T cells can be further partitioned into TSCM cells within the memory compartment and progenitor exhausted (Texh-prog) cells within the exhausted compartment (40, 41). Texh-prog cells are defined by expression of TCF1, SLAMF6, and PD-1, localize to tumors and tumor-draining lymph nodes, and retain self-renewal capacity while giving rise to more differentiated exhausted subsets (42, 43). Their abundance correlates positively with responses to immune checkpoint blockade, underscoring their central role in determining immunotherapeutic efficacy (42, 43).

### 2.3 Extension to CD4 T Cells

While stemness is well established in CD8 T cells, its extension to CD4 T cells is more recent and inherently more complex, reflecting the greater functional heterogeneity of the CD4 compartment. Unlike CD8 T cells, CD4 T cells do not follow a uniform exhaustion program but instead differentiate into diverse states, including helper, regulatory, and dysfunctional phenotypes (16, 44).

Early evidence for stem-like properties in CD4 T cells derives from studies of TSCM, identified in both CD4 and CD8 compartments (35, 36). CD4 TSCM cells comprise ~2–3% of circulating T cells, exhibit a naïve-like phenotype, and express markers such as CD95, IL-2Rβ, CXCR3, and LFA1, while retaining self-renewal capacity and the ability to regenerate diverse memory and effector subsets (36, 45). However, the broader concept of antigen-experienced stem-like CD4 T cells in disease contexts has only recently emerged.

Early evidence arose from Mycobacterium tuberculosis infection, in which PD-1^+^KLRG1^-^ CD4 T cells self-renewed and generated KLRG1^+^ TH1 effectors with superior protective capacity (46, 47). TCF1^+^ CD4 T cells with stem-like features were subsequently identified in chronic LCMV infection, inflammatory bowel disease, autoimmune vasculitis, experimental autoimmune encephalomyelitis, type 1 diabetes, transplantation, allergic disease, and cancer (18, 22, 48–53). Recent human ulcerative colitis and rheumatoid arthritis studies further resolved self-renewing CD4 progenitors clonally linked to inflammatory effectors, while a chronic graft-versus-host disease model identified Ly108^+^CD69^-^ stem-like memory CD4 T cells that replenish pathogenic tissue-resident populations (54–56). Together, these studies broaden the contexts in which stem-like CD4 states have been observed, but they do not establish that all such populations are phenotypically or functionally identical.

The identification of stem-like CD4 T cells across diverse disease contexts raises a central question: how does the tumor microenvironment (TME) shape their fate decisions, and with what consequences for antitumor immunity? Their capacity to adopt distinct effector programs across settings—favoring iTreg differentiation in tumors, generating pathogenic effectors in autoimmunity, and giving rise to TH1 and Tfh cells during infection—indicates pronounced context-dependent plasticity. This has been conceptualized as “clonal adaptation” (16), whereby stem-like CD4 T cells lack a fixed effector program and instead dynamically adjust differentiation in response to local cues. However, this model requires rigorous validation, particularly through *in vivo* lineage-tracing approaches (e.g., Cre–loxP systems), to distinguish true multipotency from pre-committed progenitor states.

### 2.4 Stem-like versus progenitor states

The application of “stemness” to T cells remains debated due to definitional imprecision. Adopting a hematopoietic framework allows discrimination between truly stem-like populations and progenitors (32, 57). Naïve CD4 T cells most closely approximate bona fide stem cells, characterized by quiescence, longevity, residence in defined niches (secondary lymphoid organs), and maximal developmental plasticity, with the capacity to generate all CD4 lineages (57, 58). TSCM and T central memory (Tcm) cells also exhibit key stem-like features, including self-renewal and multipotency. In contrast, effector memory (Tem) and tissue-resident memory (Trm) cells, despite their persistence, display more restricted lineage potential and are more appropriately classified as progenitors (58).

For the antigen-experienced TCF1^+^ lin^-^ CD4 populations considered here, the operational designation “stem-like” is supported by population-level persistence, an incompletely committed transcriptional state, and multilineage output in transfer or perturbation studies (18, 52). These observations do not establish that one individual cell both self-renews durably and generates multiple fates. The candidate populations may therefore contain true multipotent cells together with lineage-biased progenitors, a distinction that requires single-cell lineage tracing.

## 3 Molecular characteristics of stem-like CD4 T cells

### 3.1 Phenotypic definition and identification

**Figure 1:**
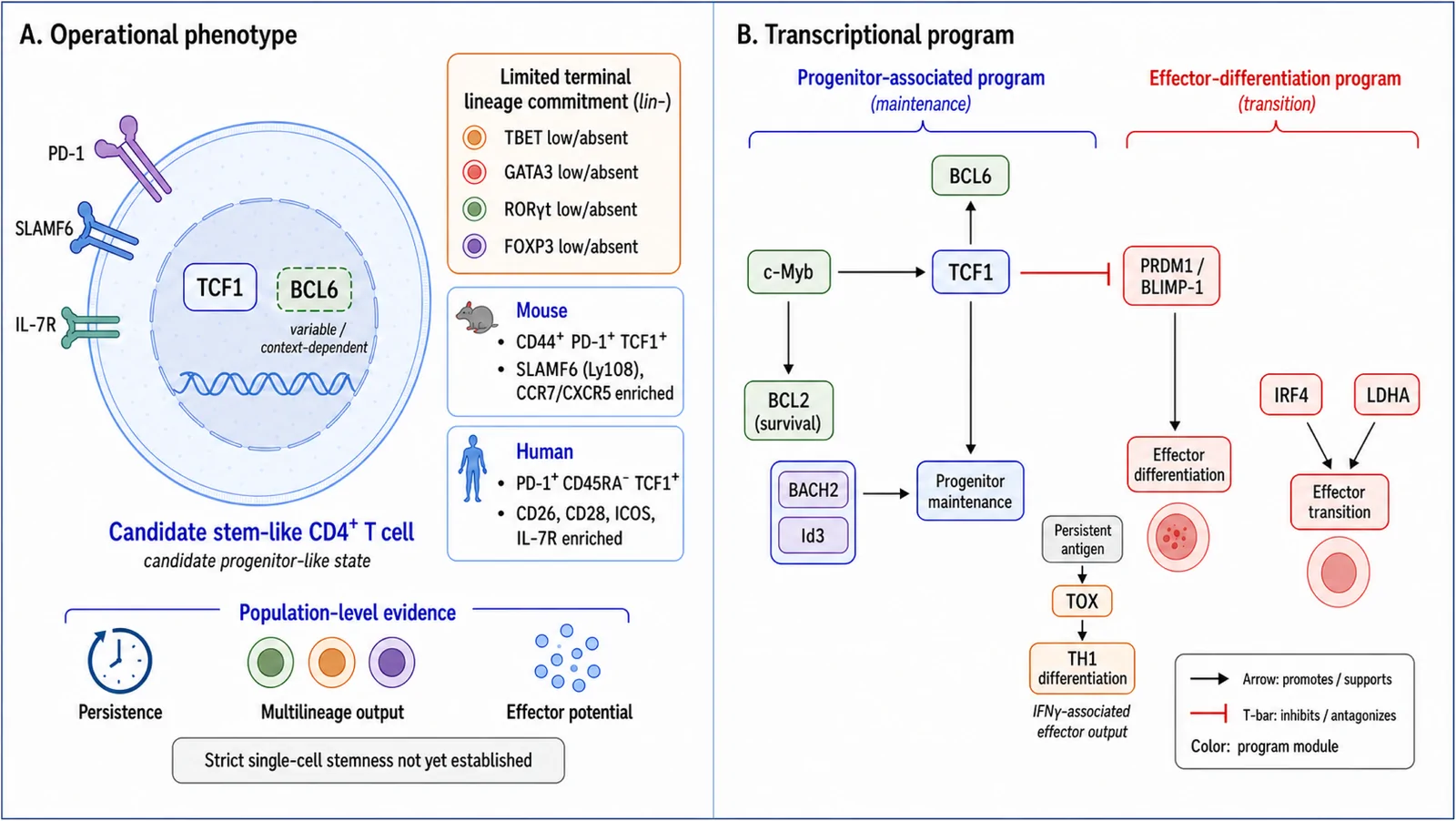
Phenotypic and transcriptional features of candidate stem-like CD4⁺ T cells. **(A)** Operational phenotype. Candidate stem-like CD4⁺ T cells are operationally identified as antigen-experienced TCF1⁺PD-1⁺ populations with limited terminal lineage commitment and population-level evidence of persistence and multilineage output; durable self-renewal and multipotent differentiation in the same individual tumor-associated CD4 T cell remain unproven. In reported murine models, the operational lin⁻ phenotype includes low or absent TBET, GATA3, RORγt, and FOXP3, whereas BCL6 is variable and may coexist with TCF1. Reported human populations show a CD45RA⁻ memory-like phenotype and enrichment of costimulatory or survival-associated molecules such as CD26, CD28, ICOS, and IL-7R. **(B)** Transcriptional program. At the molecular level, a TCF1-associated progenitor program involving BCL6, BACH2, Id3, and c-Myb restrains terminal differentiation, partly through antagonism with PRDM1/BLIMP-1. TOX has cell-type-specific effects: current CD4 evidence supports TH1 effector differentiation rather than maintenance of the early progenitor-like state. IRF4 and LDHA-associated metabolic reprogramming promote effector transition. The c-Myb–TCF1/BCL2 and TCF1/BCL6 relationships, the BACH2/Id3-associated progenitor-maintenance module, the persistent antigen–TOX effector-differentiation module, and the IRF4- and LDHA-associated effector-transition modules are related but should not be interpreted as a single linear pathway. Activation arrows and inhibitory bars denote regulatory relationships; transcription factors are shown as intracellular or nuclear regulators. Black arrows indicate context-supported promotion or support and do not necessarily imply direct, cell-intrinsic regulation in tumor-associated candidate stem-like CD4 T cells. Evidence for the c-Myb–TCF1/BCL2 relationships, BACH2/Id3-associated progenitor maintenance, and IRF4/LDHA-associated effector transition is derived in part from chronic-infection, cross-lineage, or broader T-cell studies rather than direct studies of the endogenous tumor-associated candidate population.

Stem-like CD4 T cells cannot be identified by a single universal surface marker. Operational assignment requires concordance among an antigen-experienced TCF1^+^PD-1^+^ phenotype, limited terminal lineage commitment, and population-level evidence of persistence or multilineage output (18, 22, 52). This assignment identifies a candidate progenitor-like state; it does not by itself establish strict functional stemness at the single-cell level. Murine and human criteria, together with their evidence boundaries, are summarized in **Table 1**. The phenotypic and transcriptional framework is summarized in **Figure 1**.

**Table 1:** Phenotypic and functional criteria for identifying stem-like cd4 t cells in murine and human settings.

Feature	Murine evidence	Human evidence
Core phenotype	CD44^+^PD-1^+^TCF1^+^ antigen-experienced cells	PD-1^+^CD45RA^-^TCF1^+^ antigen-experienced cells
Operational lin^-^ definition	No canonical terminal commitment to TH1, TH2, TH17, or Treg programs (TBET^-^GATA3^-^RORγt^-^FOXP3^-^); BCL6 is variable and context dependent	No canonical lineage-defining program in the assayed panel; BCL6 is not a universal exclusion marker
Enriched markers	SLAMF6 (Ly108), CD73, FR4, GPR183, ID3, CCR7, and CXCR5	CD26, CD28, ICOS, IL-7R, CCR7, and CXCR5; SLAMF6 requires broader validation
Low terminal-effector features	Low CXCR6, CD39, CTLA-4, PSGL1, SLAMF1, BLIMP-1, and CD38	Low CD39 and terminal effector molecules in reported cohorts; panels vary by study
Functional support	Population-level persistence and multilineage output supported by transfer and perturbation studies; clonotype overlap does not establish directionality or single-cell multipotency	Ex vivo proliferation and multilineage differentiation reported; *in vivo* single-cell lineage tracing is not available
Evidence boundary	Evidence spans selected tumor and non-tumor models, but durable self-renewal and multipotent output in the same individual cell remain unproven	Reported mainly in renal, bladder, and prostate cancers; pan-cancer conservation and strict functional stemness remain provisional

In murine tumor and chronic-antigen models, stem-like CD4 T cells are operationally enriched as CD44^+^PD-1^+^TCF1^+^ cells that lack canonical terminal commitment to TH1, TH2, TH17, and Treg fates, as indicated by low or absent TBET, GATA3, RORγt, and FOXP3 (18, 52). BCL6 should not be treated as a universal exclusion marker. TCF1^+^ progenitor-like CD4 cells can coexpress BCL6, and BCL6 is required for maintenance of progenitor output in persistent-antigen settings (22, 59). Thus, lin^-^ denotes the absence of fixed terminal lineage commitment rather than the absence of every transcription factor associated with a differentiated lineage.

In human renal, bladder, and prostate tumors, the corresponding population has been identified as PD-1^+^CD45RA^-^TCF1^+^ antigen-experienced cells without a dominant terminal lineage program (18). Reported cells express costimulatory and survival-associated molecules, including CD26, CD28, ICOS, and IL-7R, while showing relatively low terminal-effector features. Pan-cancer single-cell atlases reveal related TCF7-high, effector-low CD4 states, but marker panels and functional validation vary across cohorts (19). Cross-species conservation is therefore plausible but should not be interpreted as complete phenotypic equivalence.

Among these markers, SLAMF6 (Ly108) is notable because it is enriched in several progenitor or stem-like T cell states (42, 50, 52, 60). Earlier loss-of-function studies and a recent mechanistic analysis showed that disrupting SLAMF6 signaling can enhance T cell activation, reduce exhausted-cell accumulation, and improve tumor control (60–62). The recent study identified inhibitory cis homotypic SLAMF6 interactions and demonstrated activity of blocking antibodies in human T cells and tumor models. However, these data do not show that SLAMF6 blockade directly expands or preserves tumor-specific stem-like CD4 T cells. In this review, SLAMF6 is therefore treated as both a marker and a candidate regulatory receptor whose CD4-specific effect on self-renewal versus effector differentiation remains to be tested.

### 3.2 Transcriptional regulation

The transcriptional program of stem-like CD4 T cells is controlled by a network of factors that restrain terminal differentiation while preserving future lineage potential.

TCF1 (encoded by Tcf7) is a central regulator of the progenitor-like state and a major effector of canonical Wnt signaling (63–65). TCF1 can promote BCL6 expression, and the two factors cooperate to preserve self-renewal and limit terminal differentiation (66). TCF1 also supports EOMES and c-Myb programs, with c-Myb promoting survival through BCL2 (67, 68). In contrast, TCF1 represses PRDM1, which encodes BLIMP-1; rising BLIMP-1 activity is associated with loss of Cxcr5, Sell, and Ccr7 and progression toward effector differentiation (68). This relationship is therefore best represented as antagonism between the TCF1-associated progenitor program and the BLIMP-1-associated effector program, not as simultaneous activation and repression of BLIMP-1 by TCF1. Conditional Tcf7 deletion impairs persistence across chronic immune settings (18, 52, 69).

BCL6 has a dual interpretive role. It is a defining regulator of Tfh differentiation, but it also participates in a broader progenitor-maintenance program. During chronic LCMV infection, BCL6-dependent TCF1^+^ CD4 progenitors sustain both Tfh and non-Tfh effector output, and loss of Bcl6 reduces this reservoir (22, 59). BCL6 expression therefore does not by itself establish Tfh commitment, and its level may vary across tumor, infection, and autoimmune contexts. This distinction reconciles BCL6 coexpression with the use of lin^-^ to denote absence of terminal lineage fixation.

BACH2 limits terminal differentiation by restricting chromatin accessibility at sites bound by effector transcription factors such as IRF4 and AP-1, while preserving accessibility at memory-associated genes, including *Cxcr5 (*70, 71*).* In TH17 cells, BACH2 maintains a stem-like, non-pathogenic state and suppresses the pathogenic program (72). Consistently, BACH2 overexpression promotes regulatory gene accessibility, whereas its deficiency exacerbates disease in experimental autoimmune encephalomyelitis; in humans, protective variants in multiple sclerosis are associated with increased BACH2 expression in CD4^+^ T cells (72).

Id3 is required for the maintenance of TCF1^+^ stem-like CD4 T cells. In graft-versus-host disease models, *Id3* deficiency leads to increased PD-1 expression, impaired TH1 function, and reduced accumulation of TCF1^+^ cells in tissues (73). Mechanistically, Id3 preserves stemness by limiting chromatin accessibility at loci encoding factors that drive PD-1 expression and effector differentiation (73).

c-Myb acts upstream of this program by promoting *Tcf7* transcription and repressing *Zeb2*, thereby supporting stem-like properties while constraining effector differentiation (74).

TOX is induced by persistent antigen stimulation and has cell-type-specific consequences. In CD8 T cells, it supports exhaustion-associated epigenetic programs while preserving the progenitor-exhausted pool (75–78). Recent gain- and loss-of-function experiments show that TOX promotes CD4 TH1 fate commitment, IFN-γ production, cytotoxicity, and antitumor activity; the same program is associated with pathogenic autoimmune responses (79). Thus, TOX should not be interpreted as a universal marker of dysfunction. Its association with CD8 exhaustion does not transfer directly to CD4 T cells. The available CD4 evidence supports a role in effector differentiation, not maintenance of an early tumor-associated stem-like state.

IRF4 and the metabolic enzyme LDHA are linked to the transition from stem-like to effector states. In alloreactive CD4 T cells, IRF4 relieves the TCF1-associated differentiation block, in part through H3K27me3-mediated silencing of Tcf7; Irf4 loss arrests cells in a TCF1^+^ state and prevents effector generation (52). LDHA supplies the glycolytic flux required for this transition, and its loss similarly impairs effector differentiation (52). A recent transplantation study independently showed that IRF4 is required for TCF1^+^ CD8 T cells to generate KLR^+^ NK-like effectors that mediate rejection (80). This CD8 study supports a cross-lineage role for IRF4 in progenitor-to-effector progression, but it is not direct evidence for an IRF4–LDHA module in tumor-specific CD4 T cells. The proposed IRF4–LDHA axis should therefore be viewed as a mechanistically supported model that still requires CD4-specific causal testing in tumors.

### 3.3 Epigenetic programs

Stem-like CD4 T cells exhibit a distinct epigenetic landscape that enables both self-renewal and flexible lineage differentiation (81). Chromatin accessibility analyses using ATAC-seq have shown that stem-like CD4 T cells maintain open chromatin at stemness-associated loci (e.g., *Tcf7*, *Ccr7*, *Sell*), while keeping effector gene loci in a poised but not fully activated state (82, 83).

In autoimmune CD4 T cells, partial *de novo* methylation occurs at the *Tcf7* locus. This methylation is preprogrammed in circulating cells and results in reduced, yet sustained, TCF1 expression in tissue-infiltrating stem-like cells (51). Such gradual epigenetic regulation allows stem-like CD4 T cells to balance self-renewal with the capacity for effector differentiation under appropriate conditions. In TH17 cells, genome-wide comparisons of H3K4 and H3K27 trimethylation between naïve and differentiated subsets reveal greater epigenetic plasticity relative to TH1 and TH2 cells, consistent with the enhanced developmental flexibility observed in stem-like TH17 populations (84, 85).

Whether similar epigenetic mechanisms operate in tumor-specific stem-like CD4 T cells remains an important question for future investigation. Understanding how the TME shapes the epigenetic landscape of these cells may uncover new therapeutic opportunities to reprogram their differentiation fate.

### 3.4 Metabolic features

Stem-like T cells generally maintain greater mitochondrial fitness and rely more heavily on oxidative phosphorylation (OXPHOS), whereas rapidly proliferating effectors increase aerobic glycolysis to support biosynthesis and cytokine production (86–89). These programs are not fixed identities: metabolic capacity must remain sufficiently flexible for a stem-like cell to self-renew and later execute an effector transition.

Within the TH17 compartment, SLAMF6^+^ stem-like cells show lower anabolic activity and greater OXPHOS dependence than glycolytic CXCR6^+^ pathogenic effectors. Mechanistic target of rapamycin complex 1 (mTORC1) deletion prevents this metabolic transition and suppresses disease (50). This example links lineage progression to metabolic reprogramming but does not establish that OXPHOS alone creates or preserves stemness.

Cytokines and redox state further tune this balance. IL-2 favors glycolysis and terminal differentiation, whereas IL-21 sustains OXPHOS and a TSCM-like phenotype (90, 91). Limiting reactive oxygen species during early activation can also preserve less differentiated states, and progenitor exhausted CD8 T cells retain greater mitochondrial fitness than terminally exhausted cells (92, 93). Most of these mechanistic data derive from CD8 or autoimmune models and should be extrapolated to tumor-specific CD4 T cells cautiously.

Glucose deprivation and lactate accumulation exert distinct, time-dependent pressures in tumors. Reduced glucose availability can attenuate PI3K–AKT–mTOR signaling and LDHA-dependent glycolytic flux, thereby delaying the metabolic transition needed for effector differentiation (52, 87, 94, 95). This delay may transiently retain a TCF1-high phenotype, but it also limits proliferation, cytokine production, and the ability to replenish effectors. Lactate accumulation and extracellular acidification create an additional constraint by impairing glycolytic flux and mitochondrial function; prolonged hypoxia and nutrient stress can ultimately erode self-renewal and survival (87, 93, 96). A nutrient-limited environment should therefore not be equated with stemness. Direct measurements of substrate use, flux, and fate in tumor-specific stem-like CD4 T cells remain a major evidence gap.

### 3.5 Comparison with stem-like CD8 T cells

Candidate stem-like CD4 populations and progenitor-exhausted CD8 T cells (TPEX) share TCF1 expression, lymphoid localization, population-level persistence, and the capacity to generate differentiated progeny (63, 97–99). Their developmental scope differs: TPEX cells remain within the exhausted CD8 lineage, whereas candidate stem-like CD4 populations show context-dependent multilineage output at the population level (16, 20, 99–101). PD-1 blockade expands and differentiates TPEX cells but has not consistently redirected the candidate CD4 state in the tumor models examined (18, 43, 102, 103). **Table 2** therefore compares candidate stem-like CD4 cells specifically with TPEX cells; T stem cell memory (TSCM) cells are a distinct memory population discussed separately in the therapeutic section.

**Table 2:** Comparison of stem-like CD4 T cells and progenitor-exhausted CD8 T cells.

Comparison dimension	Candidate stem-like CD4 T cells	Progenitor-exhausted CD8 T cells (TPEX)
Core markers	TCF1^+^, PD-1^+^, SLAMF6-enriched, limited terminal lineage commitment; BCL6 variable	TCF1^+^, PD-1^+^, SLAMF6^+^ within the exhausted lineage
Differentiation potential	Population-level output into TH1, iTreg, Tfh, and other context-dependent states; single-cell multipotency unproven	Primarily generates intermediate and terminally exhausted CD8 states
Response to PD-1 blockade	No consistent direct fate redirection demonstrated in the tumor models examined	Expands and differentiates in response to PD-1/PD-L1 blockade
Functional role	Candidate upstream helper compartment with direct and CD8-supporting effector potential	Progenitor reservoir for exhausted-lineage responses; differentiated progeny provide cytotoxic output
Dysfunction characteristics	Contextually restrained differentiation rather than a defined canonical exhaustion continuum	Canonical progenitor-to-terminal exhaustion continuum
Tissue localization	Enriched in tumor-draining lymph nodes in selected models, with lower frequencies in tumors	Found in tumor-draining lymph nodes and intratumoral immune niches

## 4 Differentiation dynamics in tumors

This section synthesizes recent experimental evidence to develop a framework linking fate decisions of stem-like CD4 T cells to the regulation of antitumor immunity.

### 4.1 An abundant activated subset in selected tumors

The most detailed direct characterization of endogenous tumor-associated TCF1^+^PD-1^+^ lin^-^ CD4 T cells currently comes from a study that examined selected human urological tumors together with several mouse models (18). Single-cell RNA sequencing of PD-1^+^ CD4 T cells from human renal tumors identified FOXP3^+^ Treg, EOMES^+^ cytotoxic-like, and TCF7-high lineage-uncommitted clusters. Within the renal cell carcinoma (n=125), bladder cancer (n=17), and prostate cancer (n=6) cohorts analyzed in that study, the TCF1^+^ lin^-^ population was an abundant activated (PD-1^+^CD45RA^-^) subset, accounting for approximately 35% of this compartment in most patients (18). This result should not be generalized to all tumors without functional validation.

Beyond these urological tumors, pan-cancer single-cell atlases contain transcriptionally related TCF7-high, effector-low CD4 states in melanoma, lung cancer, colorectal cancer, and other solid tumors (19). These atlases do not uniformly apply the same marker panel or test persistence and multilineage output. They therefore support the possibility of cross-cancer recurrence, not functional equivalence or a universally conserved tumor CD4 state.

In murine tumor models (TRAMPC1-GP, B16F10-GP, MC38, and RENCA-HA), antigen-specific CD4 T cells identified by MHC-II tetramers rapidly acquire a TCF1^+^ lin^-^ phenotype in tumor-draining lymph nodes (TDLNs) within one week of tumor inoculation (18). By week five, most tumor-specific CD4 T cells in TDLNs retain this stem-like state, with a minority expressing FOXP3 or BCL6 and few expressing TBET, RORγt, or EOMES. In contrast, tumor-infiltrating CD4 T cells are enriched for Treg cells, with only a small fraction maintaining the TCF1^+^ lin^-^ phenotype (18). This spatial distribution—stem-like cells predominating in TDLNs and Treg cells within tumors—suggests that TDLNs function as a primary reservoir and site of fate determination for stem-like CD4 T cells.

Adoptive transfer of SMARTA TCR transgenic CD4 T cells (LCMV glycoprotein–specific) supports this trajectory: naïve cells transition to a TCF1^+^ lin^-^ state in TDLNs within 7 days, and over 4–5 weeks, ~20% of TDLN-resident cells and ~85% of tumor-infiltrating cells acquire FOXP3 expression (18). These data indicate that tumor-specific CD4 T cells are initially programmed into a stem-like state in TDLNs and subsequently undergo progressive differentiation toward an iTreg fate within the tumor microenvironment.

### 4.2 The restrained state: Treg-mediated suppression

A central finding is that stem-like CD4 T cells in tumors exist in a “restrained” state—competent for effector differentiation but actively suppressed by Treg cells and the tumor microenvironment (18).

First, functional assays demonstrate that TCF1^+^ lin^-^ CD4 T cells isolated from human renal tumors proliferate robustly upon stimulation, whereas CD39^+^ effector CD4 T cells from the same tumors remain largely non-proliferative (18). Under polarizing conditions, TCF1^+^ lin^-^ cells differentiate into TH1 (TBET^+^), Treg (FOXP3^+^), Tfh (BCL6^+^), and EOMES^+^ lineages, whereas CD39^+^ cells remain terminally committed (18). These data support a substantial contribution of extrinsic suppression to impaired effector differentiation *in vivo*, without excluding reversible cell-intrinsic constraints.

Second, PD-1/PD-L1 blockade does not substantially alter CD4 T cell fate. Following anti–PD-L1 treatment, antigen-specific and total PD-1^+^ CD4 T cells retain stem-like and Treg phenotypes in both resistant and responsive tumor models (18), in contrast to its established effects on CD8 T cells (43, 102). This suggests that PD-1 is not the dominant regulator of CD4 T cell differentiation in tumors.

Third, Treg depletion releases this restraint. In DEREG mice, transient depletion of FOXP3^+^ Treg cells induces expansion and TH1 differentiation of tumor-specific CD4 T cells in both TDLNs and tumors, characterized by downregulation of TCF1 and upregulation of TBET, effector markers, and cytokine production (IFN-γ, IL-2) (18). This transition peaks within days and persists after Treg reconstitution, indicating a self-sustaining differentiation program (18).

TCRβ clonotype overlap is consistent with developmental relatedness but does not establish directionality. Under baseline conditions, candidate stem-like CD4 populations predominantly share clonotypes with Treg and Tfh populations; after Treg depletion, more than 90% of clonotypes in the expanded TH1 cluster overlap with the candidate progenitor pool (18). Adoptive transfer of sorted SMARTA TCF1^+^ lin^-^ CD4 T cells into Treg-depleted tumor-bearing hosts further supports a population-level precursor–product relationship by showing expansion, persistence, and preferential TH1 output (18). Neither clonotype overlap nor bulk transfer proves that one cell both self-renews durably and generates multiple fates.

However, Treg depletion does not definitively establish Tregs as the sole mediators of suppression. Alternative explanations include shifts in the inflammatory milieu (e.g., increased IL-12), concurrent modulation of other immunosuppressive populations, including myeloid-derived suppressor cells (MDSCs) and tumor-associated macrophages (TAMs), and temporal effects, whereby the peak TH1 response reflects secondary immune activation rather than direct release from inhibition. Accordingly, the “restrained” state likely reflects a composite regulatory network involving Treg cells, cytokine signals, metabolic constraints, and epigenetic control.

### 4.3 Working model: TH1 differentiation and TDLN-centered CD4–CD8 cooperation

The transition from a restrained to an activated state may contribute to antitumor immunity through a tumor-draining lymph node (TDLN)-centered CD4–CD8 cooperative axis. We use the term working model because the spatial sequence and the direct cellular target of CD4-derived IFN-γ have not yet been established.

Following Treg depletion, stem-like CD4 T cells differentiate into IFN-γ-producing TH1 effectors (18). In the proposed model, CD4-derived IFN-γ contributes to the differentiation of TCF1^+^ stem-like CD8 T cells in TDLNs, after which CD8 effectors enter tumors. The available experiments support temporal coupling between these events but do not yet distinguish direct IFN-γ signaling in CD8 T cells from indirect effects mediated by dendritic or stromal cells.

This model shares features with the classical CD4 help paradigm but also differs mechanistically. In the canonical framework, CD4 T cells license dendritic cells via CD40L–CD40 interactions, enabling antigen cross-presentation and cytokine production (e.g., IL-12) that support CD8 T cell responses (104–106). In tumors, IFN-γ produced by TH1-differentiated CD4 T cells has been proposed to promote CD8 effector differentiation (18). However, direct evidence that IFN-γ acts independently of dendritic cell licensing remains limited. Key validation studies are required, including spatial analyses to establish proximity between TH1 and stem-like CD8 T cells in TDLNs, assessment of IFN-γ receptor signaling in these cells, cytokine neutralization experiments, and dendritic cell perturbation approaches. Accordingly, the CD4–CD8 cooperative axis should be considered a working model. Dendritic cells likely remain central to antigen presentation and costimulation, with IFN-γ potentially acting indirectly through modulation of dendritic cell function rather than bypassing these pathways.

In addition to supporting CD8 responses, CD4 T cells can exert direct antitumor activity. In the Trp1 melanoma system, tyrosinase-related protein 1–specific CD4 T cells first occupy a TCF1^+^Ly108^+^ stem-like state and then generate TCF1^-^CXCR6^+^ TH1-like effectors. These effectors support tumor control, although the mechanism of tumor elimination remains unresolved and may be either tumor-cell MHC class II dependent or independent. They are short-lived and require replenishment from the progenitor pool. LDHA-dependent aerobic glycolysis is required for this transition; LDHA deletion blocks effector generation and antitumor activity (107). This model directly links metabolic reprogramming to CD4 effector output.

Taken together, these findings support a provisional framework in which CD4 fate decisions in TDLNs influence downstream CD8 responses (108, 109). Treg cells may inhibit this process both by directly suppressing CD8 T cells and by preventing stem-like CD4 T cells from producing TH1 help (18, 110). The relative contribution of direct CD4–CD8 communication, dendritic-cell licensing, and broader inflammatory remodeling remains unresolved.

Enforced TBET expression provides proof of principle that CD4 fate can be redirected: in adoptive models it promotes TH1 differentiation, restores CD8 effector generation, and improves tumor control despite Treg-mediated restraint (18). Because ectopic TBET can bypass physiological regulatory steps, this result does not establish that endogenous CD4 fate is the sole rate-limiting determinant of antitumor immunity.

### 4.4 Multipotency and context-dependent fate

A defining feature of stem-like CD4 T cells is their context-dependent differentiation capacity, termed “clonal adaptation” (16). In transfer experiments, tumor-derived stem-like CD4 T cells from TDLNs, when introduced into naïve hosts and challenged with LCMV Armstrong, underwent >20-fold expansion and differentiated into TH1 and Tfh cells at frequencies comparable to endogenous virus-specific CD4 T cells, with minimal Treg conversion (18). In contrast, Treg cells isolated from the same tumors maintained stable FOXP3 expression across conditions (18).

Environmental changes can alter CD4 differentiation outcomes, but the relevant cellular actors are not equivalent across models. Across separate disease contexts, phenotypically related candidate populations generate TH1 and Tfh cells during chronic infection, pathogenic TH17 cells in autoimmunity, and TH2 effectors in allergic disease; in a distinct tolerance setting, Thetis cell IV antigen-presenting cells instruct food-specific peripheral Treg differentiation (50, 53, 111, 112). Together, these studies illustrate context-dependent CD4 fate regulation, but the Thetis-cell study does not establish a stem-like CD4 precursor population, and the cross-study pattern does not show that one cell retains all of these fates or that the populations are biologically equivalent.

This concept has been extended to the TH2 lineage. In human allergic disease, TCF1^+^LEF1^+^ TH2 multipotent progenitors (TH2-MPP) show persistence and give rise to TH2 effector, Tfh, and Treg populations, with developmental relatedness supported by TCR clonotype analysis (53). These cells are resistant to IL-4R blockade but responsive to TSLP (53). HIF2α promotes differentiation of TCF1^+^Ly108^+^ cells into pathogenic TH2 effectors, acting with GATA3 to modulate phospholipid metabolism and enhance TCR–PI3K–AKT signaling (113). Collectively, these data support recurrent population-level multilineage potential. Single-cell multipotency and cross-disease equivalence remain unresolved.

### 4.5 Models of differentiation

A central question is whether stem-like CD4 T cells are truly multipotent or exhibit early lineage bias.

Evidence for population-level multilineage potential derives from tumor, tolerance, and infection models. Candidate stem-like CD4 cells isolated from TDLNs differentiate into TH1 and Tfh populations after transfer to infectious settings, while Thlin^-^ cells generated under tolerogenic conditions can mount effector responses after Treg depletion (18, 114). In chronic LCMV infection, related precursors also generate TH1 and Tfh populations (22, 115). These observations are consistent with common-precursor models but do not demonstrate multipotency of an individual cell.

However, several observations indicate greater complexity. Hybrid states with mixed transcriptional features have been reported (116, 117). Stem-like CD4 T cells share BCL6 dependence and CXCR5 expression with Tfh cells (20, 22), and CD62L^+^PD-1^lo memory-like Tfh cells can occupy a progenitor-like niche early in responses (118). These findings suggest heterogeneity within the stem-like pool, potentially including subsets with biased differentiation potential, consistent with a branching model. Notably, Tfh cells can convert into long-lived TH1 cells in the absence of B cells (119), indicating that such bias does not preclude plasticity.

An integrative model is that multipotency declines over time under chronic antigen exposure: early stem-like cells may be broadly multipotent, whereas later populations become progressively biased. Although this framework reconciles existing data, it remains to be directly tested by longitudinal lineage-tracing approaches.

In tumors, current evidence supports population-level plasticity more strongly than strict single-cell multipotency. TCF1^+^ lin^-^ CD4 populations lack dominant terminal-lineage markers and can adopt alternative fates after transfer into non-tumor environments (18). Whether this pool contains multipotent cells, lineage-biased progenitors, or both—and whether its potential narrows during tumor progression—requires longitudinal single-cell lineage tracing combined with multi-omics.

## 5 Tumor microenvironmental regulation

**Figure 2:**
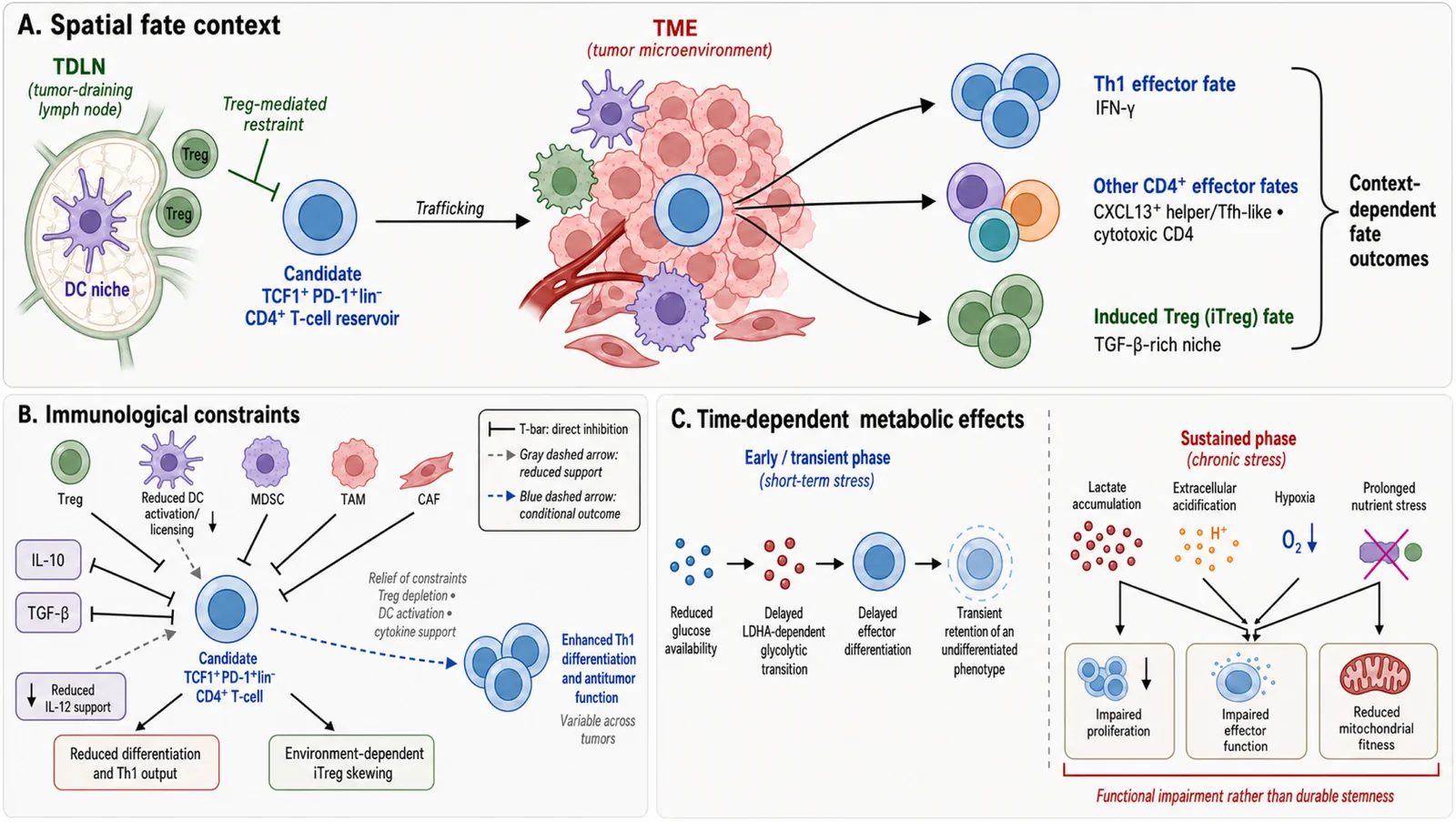
Multilevel mechanisms shaping the differentiation of candidate stem-like CD4⁺ T cells in the tumor microenvironment. **(A)** Spatial fate context. The differentiation of candidate stem-like CD4⁺ T cells is shaped by integrated spatial, immunological, and metabolic signals. **(B)** Immunological constraints. In tumor-draining lymph nodes, Treg cells, dendritic-cell function, and cytokine availability may constrain early fate decisions; within tumors, TGF-β, IL-10, myeloid and stromal cells, and metabolic competition reinforce a restrained state. **(C)** Time-dependent metabolic effects. Reduced glucose availability can delay the LDHA-dependent glycolytic transition needed for rapid effector differentiation and may transiently retain an undifferentiated phenotype, but it simultaneously limits proliferation and effector output. Lactate accumulation, extracellular acidification, hypoxia, and prolonged nutrient stress can impair both effector function and mitochondrial fitness. A metabolically restrictive environment should therefore not be equated with durable stemness. The mechanisms are separated conceptually to distinguish transient phenotypic retention from durable self-renewal. T-bars denote inhibitory influences or constraints; depending on context, the underlying effects may be direct or indirect and should not be interpreted as evidence that every depicted component directly inhibits the candidate population. Gray dashed arrows indicate reduced positive support, whereas blue dashed arrows indicate conditional outcomes after relief of constraints rather than uniform causal responses across tumors. The time-dependent metabolic panel presents a working model; direct metabolic-flux measurements in tumor-specific candidate stem-like CD4 T cells remain limited. DC, dendritic cell; MDSC, myeloid-derived suppressor cell; TAM, tumor-associated macrophage; CAF, cancer-associated fibroblast.

The TME constitutes a distinct immunological niche that shapes stem-like CD4 T cell fate differently from infection and autoimmunity. Defining these regulatory mechanisms is essential for developing strategies to direct their differentiation toward antitumor effector states (**Figure 2**).

### 5.1 Treg cells as key regulators

As outlined in Section 4, Treg cells are a principal regulator of stem-like CD4 T cell differentiation in tumors (18). This contrasts with autoimmune settings, where stem-like CD4 T cells generate pathogenic effector populations despite regulatory constraints (21, 51, 120). The bias toward iTreg differentiation in tumors likely reflects multiple factors: enrichment of Treg cells in tumors and TDLNs, creating a tolerogenic cytokine milieu (121); production of TGF-β and IL-10 by Treg cells, tumor cells, and tumor-associated macrophages, promoting FOXP3 induction (122); and relative deficiency of pro-inflammatory cues, such as IL-12 or IL-6/IL-23, that drive TH1 and TH17 differentiation (123, 124).

Notably, Treg-mediated suppression appears to act primarily within TDLNs rather than exclusively in the tumor. This spatial organization suggests that effective therapeutic strategies must target Treg-dependent regulation at the level of draining lymph nodes to redirect stem-like CD4 T cell fate (18, 108, 109).

### 5.2 Cytokine constraints on effector differentiation

In infection and autoimmunity, strong innate activation establishes cytokine environments that drive effector differentiation: dendritic cell–derived IL-12 promotes TH1 polarization, IL-6 and IL-23 support TH17 differentiation, and IL-4 from innate lymphoid cells initiates TH2 responses (125–127). In contrast, these pro-inflammatory cues are often attenuated in the TME, consistent with its immunosuppressive character (122, 128, 129).

Consistently, stem-like CD4 T cells isolated from human tumors rapidly upregulate TBET and adopt a TH1 program upon IL-12 stimulation in dendritic cell co-culture (18), indicating preserved differentiation capacity. Thus, insufficient polarizing signals likely contribute substantially to maintenance of the restrained state, while reversible cell-intrinsic constraints remain possible. This limitation may be compounded by dysfunctional dendritic cells in the TME, which can exhibit impaired antigen presentation and altered inflammatory cytokine production (130–132).

### 5.3 Spatial control: TDLNs as sites of fate determination

Emerging evidence identifies TDLNs as key sites of fate determination for stem-like CD4 T cells. In both human and murine systems, these cells are enriched in TDLNs, whereas differentiated effector and Treg populations predominate within tumors (18). This spatial organization aligns with a broader role for lymphoid niches in antitumor immunity, including impaired antigen presentation in TDLNs and localization of stem-like CD8 T cells to lymphoid tissues and niches such as tertiary lymphoid structures (132–134).

TDLNs provide a microenvironment in which stem-like CD4 T cells interact with antigen-presenting dendritic cells, Treg cells, and cytokine signals that collectively shape their differentiation trajectory. Accordingly, the functional state of TDLNs is likely a critical determinant of antitumor immunity. Consistent with this, neoadjuvant immunotherapy administered prior to tumor and lymph node resection has demonstrated clinical benefit, potentially by targeting the reservoir of stem-like T cells within intact TDLNs (108, 109).

### 5.4 Comparison with autoimmune contexts

A notable paradox emerges across disease contexts. In autoimmunity, stem-like CD4 T cells form a persistent reservoir that continuously generates pathogenic effectors and sustains disease (49). In tumors, phenotypically related candidate populations are present but remain functionally restrained, failing to produce effective antitumor responses (16, 18).

This divergence likely reflects differences in the local environment. Autoimmune settings provide strong pro-inflammatory signals—such as IL-23 in EAE or innate activation in vasculitis—that actively drive effector differentiation (49–51), whereas tumors impose a tolerogenic milieu. In addition, the Treg-to-conventional CD4 T cell ratio is typically elevated in tumors, favoring suppression (135, 136). Tissue context also differs: stem-like CD4 T cells in autoimmunity often reside within inflammatory tertiary lymphoid structures (120), whereas TLS in tumors are heterogeneous in presence and composition.

These observations suggest that converting the tumor environment from tolerogenic to inflammatory may unlock the effector potential of stem-like CD4 T cells, although such approaches require careful control to avoid autoimmunity.

Further support for environmental control comes from peripheral tolerance models. In oral tolerance, antigen-specific CD4 T cells expand as anergic-like, lineage-negative cells under Treg-mediated suppression and preferentially differentiate into Treg cells; after Treg depletion, Thlin^-^, Tfh, and TH1 populations expand markedly (114). During pregnancy, maternal CD4 T cells recognizing fetal antigens proliferate without effector differentiation yet retain functional competence when conditions change (137). Together, these findings support a substantial role for environmental cues while not excluding pre-existing lineage bias or epigenetic constraints.

### 5.5 Immunosuppressive and metabolic networks

While Treg cells are key regulators of candidate stem-like CD4 differentiation, they are not the sole source of immunosuppression in the TME. Tumor cells, stromal elements, and myeloid populations collectively constrain T-cell function through intrinsic and paracrine mechanisms (44, 128, 129). Myeloid-derived suppressor cells inhibit T-cell proliferation through arginase, nitric oxide, and reactive oxygen species; tumor-associated macrophages secrete IL-10 and TGF-β; and regulatory dendritic cells suppress activation through indoleamine 2,3-dioxygenase-mediated tryptophan depletion (138, 139). Cancer-associated fibroblasts establish physical and chemokine barriers, including TGF-β- and CXCL12-associated exclusion, that can restrict T-cell infiltration into tumor cores (140, 141). Whether these barriers directly alter CCR7/CXCR5-dependent trafficking of candidate stem-like CD4 cells has not been demonstrated. Amino-acid depletion may impair mitochondrial fitness in these cells (93, 142), and macrophage-derived IL-10 may sustain a restrained state by limiting dendritic-cell IL-12 (143, 144). These CD4-specific mechanisms remain largely hypothetical and require direct perturbation.

The TGF-β pathway likely plays a central role in regulating stem-like CD4 T cell fate. As a potent inducer of FOXP3 expression and iTreg differentiation, TGF-β is abundantly produced in the TME by Treg cells, tumor-associated macrophages, cancer-associated fibroblasts, and tumor cells, collectively promoting skewing toward the iTreg lineage (145, 146). Consistent with this, in a prostate cancer bone metastasis model, elevated TGF-β suppressed TH1 differentiation and conferred resistance to anti–CTLA-4 therapy, whereas sites with lower TGF-β levels permitted TH1 polarization and therapeutic responsiveness (147). These findings provide indirect support for the possibility that spatial heterogeneity in TGF-β availability across tumor sites may differentially shape candidate stem-like CD4 T cell fate.

The metabolically restrictive TME likely influences both maintenance and function of stem-like CD4 T cells. Their relative reliance on OXPHOS contrasts with the LDHA-dependent glycolytic shift required for rapid effector differentiation (91, 94, 95). Glucose deprivation may delay that transition, whereas lactate accumulation, acidification, and hypoxia impose additional stress that can impair both effector output and long-term mitochondrial fitness (87, 93, 95, 96). These effects are time dependent and should not be summarized as “limited environment = stemness.” Extensive metabolic differences between stem-like and effector CD4 states have been reported, but direct flux measurements in tumor-specific cells remain sparse (52). Future studies should distinguish transient preservation of an undifferentiated phenotype from durable self-renewal under chronic metabolic stress.

The gut microbiota may represent an additional regulatory layer, indirectly shaping stem-like CD4 T cell differentiation through systemic immune and metabolic signals. However, direct evidence linking microbiota to tumor-specific stem-like CD4 T cells remains limited and is not addressed in detail here.

## 6 Therapeutic implications

**Figure 3:**
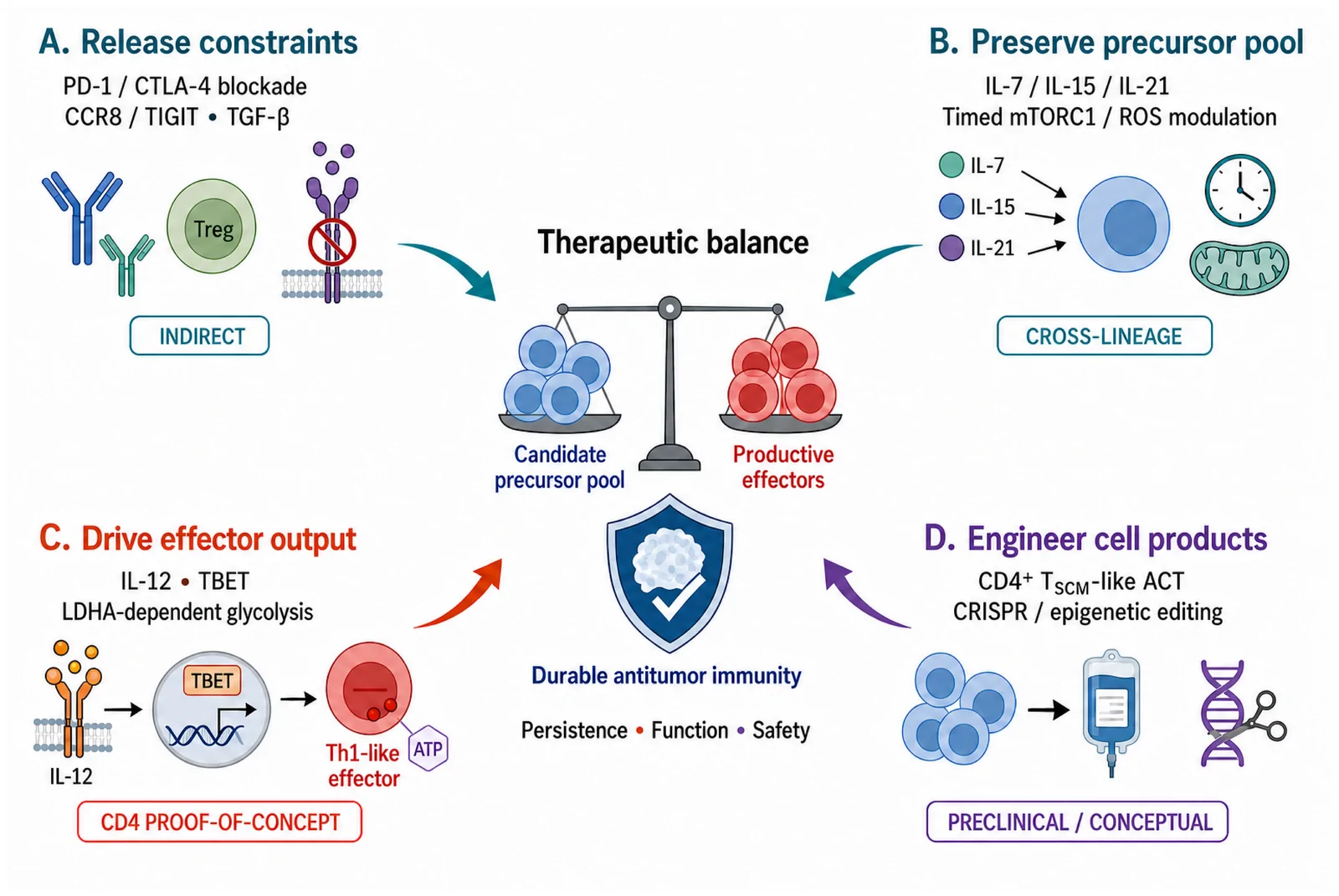
Evidence-stratified therapeutic strategies for candidate stem-like CD4^+^ T cells. The schematic organizes candidate interventions into four evidence-bounded strategy classes. **(A)** Release constraints through combined PD-1 and CTLA-4 blockade, CCR8-directed Treg targeting, TIGIT blockade, and inhibition of TGF-β signaling; evidence for effects on the endogenous candidate population is predominantly indirect. **(B)** Preserve the precursor pool through cytokine support with IL-7, IL-15, or context-dependent IL-21, or through timed modulation of mTORC1 activity or ROS; support is derived mainly from cross-lineage and ex vivo T-cell-manufacturing studies rather than direct *in vivo* studies of endogenous tumor-associated candidate stem-like CD4 T cells. **(C)** Drive productive effector output through IL-12/TBET-associated signaling and LDHA-dependent glycolysis; these represent CD4 proof-of-concept mechanisms and should not be interpreted as a single, fully validated therapeutic pathway. **(D)** Engineer cell products using CD4^+^ TSCM-like adoptive cell therapy (ACT) and CRISPR- or epigenetic-editing strategies; engineered products are distinct from the endogenous TCF1^+^PD-1^+^ lin^-^ candidate population, and the supporting evidence remains preclinical or conceptual. The evidence labels describe the proximity of supporting data to the endogenous candidate population and do not rank comparative clinical efficacy. The central balance emphasizes that durable antitumor immunity requires coordinated persistence, productive effector function, and safety. mTORC1, mechanistic target of rapamycin complex 1; ROS, reactive oxygen species; ACT, adoptive cell therapy; TSCM, T stem cell memory.

The stem-like CD4 framework suggests therapeutic opportunities beyond PD-1 monotherapy, but the evidence is uneven. Direct evidence for the endogenous tumor-associated TCF1^+^PD-1^+^ lin^-^ population remains concentrated in the models summarized in Section 4. The Trp1 system and ex vivo-generated CD4 TSCM-like adoptive products provide important but non-identical evidence, while checkpoint blockade, CAR-T persistence, cytokine manufacturing, infection, autoimmunity, transplantation, and CD8 studies are largely supportive or cross-lineage. Section 6 and **Table 3** therefore distinguish direct CD4 evidence from mechanistic inference and translational extrapolation. These evidence-bounded therapeutic concepts are summarized in **Figure 3**, which distinguishes indirect constraint-release strategies, cross-lineage precursor-preservation approaches, CD4 proof-of-concept effector-driving mechanisms, and preclinical or conceptual engineering strategies.

**Table 3:** Therapeutic strategies relevant to stem-like cd4 t-cell biology: evidence and translational boundaries.

Therapeutic strategy	Evidence specific to stem-like CD4 T cells	Key finding and limitation	Translational status
Combination ICB (anti-PD-1 + anti-CTLA-4)	Indirect clinical evidence	The combination is clinically effective, but release of endogenous stem-like CD4 differentiation through Treg depletion remains a mechanistic inference	Approved in several cancers; CD4 fate biomarkers not established
CCR8-directed Treg targeting/TIGIT blockade	Indirect mechanistic rationale; no target-specific evidence for stem-like CD4 fate redirection	CCR8-directed Treg targeting and selected Fc-engineered TIGIT-blockade platforms may reduce intratumoral Treg activity, but whether benefit is mediated by release of stem-like CD4 differentiation is unknown	Early-phase clinical development
Stemness-based ACT	Direct preclinical evidence for CD4 TSCM-like products; indirect relevance to endogenous TCF1^+^PD-1^+^lin^-^ cells	CD4 TSCM-like products can improve persistence and CD8 support, but they are not equivalent to the endogenous tumor-associated candidate population	Concept supported; product definitions require standardization
IL-7/IL-15/IL-21 culture protocols	Predominantly TSCM and CD8 evidence	Preserve less-differentiated states during manufacturing; efficacy in tumor-specific candidate stem-like CD4 cells requires direct testing	Used experimentally in ACT manufacturing
IL-12 delivery	Ex vivo differentiation evidence	Promotes TH1 differentiation, but systemic toxicity and limited *in vivo* CD4-specific evidence constrain translation	Engineered and local-delivery variants in trials
TGF-β pathway blockade	Mechanistically plausible; indirect tumor-model evidence	May reduce iTreg skewing and permit TH1 polarization, but cell-intrinsic effects are unresolved	Clinical trials ongoing in combination regimens
Forced TBET expression	Direct proof of concept in adoptive models	Can redirect fate and restore antitumor cooperation; ectopic expression may bypass physiological controls	Preclinical research
Epigenetic or metabolic modulation	Mostly cross-lineage evidence	May preserve or release less-differentiated states depending on timing; CD4-specific efficacy and Treg safety remain uncertain	Exploratory combination development
CRISPR-based fate engineering	No direct therapeutic validation	Technically feasible, but IRF4 or BLIMP-1 editing could impair necessary effector generation and requires safety testing	Conceptual/preclinical stage

ICB, immune checkpoint blockade; ACT, adoptive cell therapy; TSCM, T stem cell memory.

### 6.1 Immune checkpoint blockade beyond PD-1

PD-1/PD-L1 blockade acts prominently on stem-like CD8 T cells, promoting expansion and effector differentiation (42, 43, 102). In contrast, available tumor models have not shown consistent redirection of stem-like CD4 fate after PD-1/PD-L1 blockade (18). This difference supports combination strategies, but it does not prove that the clinical benefit of PD-1 blockade is exclusively CD8 mediated or that Treg depletion will release the same CD4 program in every tumor.

Consistent with this model, TH1 polarization of CD4 T cells in renal cancer patients is associated with improved responses to immunotherapy and prolonged progression-free survival (18). This suggests that CD4 T cell differentiation state may serve both as a biomarker of immune checkpoint blockade (ICB) responsiveness and as a therapeutic target. Patients in whom stem-like CD4 T cells can be readily directed toward a TH1 fate may derive greater benefit, whereas those with a more constrained state may require additional interventions.

Combination strategies that activate stem-like CD8 T cells while releasing or redirecting the CD4 compartment are biologically plausible. Anti-CTLA-4 antibodies can deplete intratumoral Treg cells through Fc-dependent mechanisms (148, 149), CCR8-directed Treg targeting may improve tumor specificity, whereas TIGIT blockade can affect multiple immune compartments and only selected Fc-engineered platforms preferentially deplete TIGIThi intratumoral Treg cells (150, 151). However, clinical efficacy of these agents cannot yet be attributed specifically to differentiation of stem-like CD4 T cells. Prospective studies should measure CD4 fate states before and after treatment rather than infer the mechanism from bulk Treg depletion.

In the selected tumor models analyzed by Cardenas et al., anti-PD-L1 did not substantially redirect the TCF1^+^ lin^-^ CD4 population toward alternative fates (18). This result does not mean that PD-1 blockade is functionally irrelevant to CD4 T cells: in human tumors, it can restore helper activity in exhausted PD-1-high CD39^+^ CD4 tumor-infiltrating lymphocytes (152). Fate redirection and functional rescue are therefore distinct endpoints. The apparent contrast with TPEX expansion may reflect differences in cell state, dominant Treg-mediated constraints, observation windows, or model context. PD-1 blockade alone may be insufficient to redirect the candidate progenitor-like CD4 compartment, but systematic CD4-versus-CD8 signaling comparisons are needed before drawing lineage-wide conclusions.

Distinct immune checkpoint targets exert differential effects on CD4 and CD8 T cell compartments. In human melanoma, PD-1 blockade primarily expands proliferative, exhausted-like CD8 T cells, whereas CTLA-4 blockade promotes expansion of ICOS^+^ TH1-like CD4 effector cells while also affecting subsets of exhausted CD8 T cells (153). These observations indicate that anti–CTLA-4 and anti–PD-1 therapies engage complementary immune populations, providing a mechanistic basis for combination approaches. As a third FDA-approved checkpoint, LAG-3 has not been systematically evaluated in stem-like CD4 T cells. Given its preferential interaction with MHC class II molecules, it may play a distinct role in regulating CD4 T cell function (154).

Emerging clinical data support a central role for CD4 effector subsets in responses to immune checkpoint blockade. In hepatocellular carcinoma, CXCL13^+^ CD4^+^ helper T cells are enriched in tumors from responders to anti–PD-1 therapy and are associated with enhanced CD8 T cell expansion and effector differentiation (155). In bladder cancer, responses to anti–PD-L1 therapy correlate with the presence of intratumoral cytotoxic CD4 T cells rather than CD8 T cells (156). These cells undergo clonal expansion, mediate MHC class II–restricted tumor killing, and associate with improved clinical outcomes (156). Together, these findings suggest that differentiation of stem-like CD4 T cells into distinct effector subsets—including TH1, CXCL13^+^ helper, and cytotoxic CD4 populations—may contribute to multiple mechanisms of response to checkpoint blockade. Systematic evaluation of CD4 effector subsets as predictive biomarkers warrants further investigation.

### 6.2 Adoptive cell therapy and stemness preservation

The population-level persistence and differentiation capacity associated with TCF1^+^lin^-^ CD4 cells make them candidates for adoptive cell therapy. Conventional tumor-infiltrating lymphocyte and CAR-T products can be limited by poor engraftment, terminal differentiation, and loss of persistence (157, 158). Whether prospective purification of the endogenous tumor-associated population is superior to manufacturing a broader less-differentiated T-cell product remains unknown.

Brightman et al. showed that adoptive transfer of neoantigen-specific CD4 T cells differentiated ex vivo into a TSCM-like state with IL-7 and IL-15 enhances antitumor efficacy in MHC class II–deficient squamous cell carcinoma (159). These cells proliferate *in vivo*, generate effector-memory populations in tumors, and expand PD-1^+^ CD8 T cells in draining lymph nodes (159). This is direct preclinical evidence for an engineered CD4 TSCM-like product, not proof that the product is equivalent to the endogenous TCF1^+^PD-1^+^lin^-^ population described in selected tumors.

Cytokine-based manufacturing can enrich less-differentiated T cell products. IL-15 favors memory-like metabolic states compared with IL-2 (160, 161), while IL-7 plus IL-15 supports TSCM generation and expansion (36, 162). IL-21 is context dependent: in CD8 T-cell studies, it can limit terminal differentiation and preserve stemness- and OXPHOS-associated features, whereas IL-21R signaling can promote effector polarization in other CD4 contexts, including Th2 responses (91, 163). IL-7/IL-15 protocols have been incorporated into CAR-T manufacturing (164). A recent first-in-human comparison showed that donor-derived CAR-modified TSCM cells expanded more strongly, persisted longer, and reconstituted a stem-like compartment more effectively than standard CAR-T cells (165). The product included T cells rather than a purified stem-like CD4 subset, so the study supports the manufacturing principle but does not establish a CD4-specific mechanism.

Clinical observations nevertheless support durable contributions from CD4 CAR-T cells. In two patients with chronic lymphocytic leukemia, decade-long remission was accompanied by long-term persistence of proliferative and cytotoxic CD4^+^ CAR-T clones (166). Separately, CAR TSCM cells showed superior expansion and persistence relative to conventional donor-derived CAR-T products (165). These studies establish that durable, less-differentiated T cell products are clinically feasible. They do not show that the TCF1^+^PD-1^+^lin^-^ population defined in tumors is the same population responsible for long-term CAR-T persistence.

### 6.3 Fate reprogramming strategies

A key translational implication is that the differentiation fate of stem-like CD4 T cells is not fixed but can be actively redirected. Functional studies show that enforced TBET expression is sufficient to reprogram these cells within a constrained trajectory, restoring CD8 T cell effector differentiation and enhancing responses to immunotherapy (18), establishing that fate is manipulable. However, these findings are largely derived from adoptive transfer systems, and their relevance to endogenous cells under physiological conditions remains to be established. In addition, ectopic transcription factor expression may bypass native epigenetic constraints and therefore may not fully recapitulate physiological mechanisms of releasing the restrained state.

Based on this framework, candidate stem-like CD4 fate can be manipulated at several levels. At the transcriptional level, enhancing TBET activity or inhibiting TGF-β signaling may promote TH1 differentiation, although direct evidence in endogenous tumor-associated cells remains limited. At the cytokine level, IL-12 drives TH1 polarization (18), while engineered IL-12 variants aim to reduce systemic toxicity. Metabolic interventions may alter the balance between persistence and effector output: mTORC1 inhibition can enrich less-differentiated states (167), and early suppression of reactive oxygen species expands TSCM-like pools (95), although most evidence is CD8-derived. In CD8 T cells, DNMT, HDAC, and EZH2 inhibitors can enhance progenitor-associated programs (168, 169), but CD4-specific efficacy and effects on Treg stability remain unresolved. CRISPR or epigenetic editing of IRF4, BLIMP-1, TCF1, BCL6, or BACH2 is technically feasible, yet such interventions could also impair necessary effector generation. Systematic optimization and safety testing in endogenous tumor-specific CD4 populations are therefore required.

Collectively, transcriptional, cytokine, metabolic, epigenetic, and genetic interventions offer ways to manipulate the balance between persistence and effector differentiation. Only a subset has been tested directly in candidate stem-like CD4 cells, and fewer still in endogenous tumor-specific populations. The translational objective is not simply to maximize stemness or effector differentiation, but to generate sufficient effector output while preserving a candidate precursor compartment and avoiding systemic autoimmunity.

### 6.4 Biomarkers and patient stratification

The stem-like CD4 T cell framework provides opportunities for patient stratification and biomarker development. The association between TH1 polarization and improved responses to immune checkpoint blockade and progression-free survival in renal cancer (18) suggests that profiling CD4 T cell differentiation states—particularly the balance among stem-like, TH1, and Treg subsets—may refine patient selection.

Potential biomarkers include: (i) the frequency of TCF1^+^ lin^-^ CD4 T cells in tumors or TDLNs; (ii) the TH1-to-Treg ratio among tumor-specific CD4 T cells; (iii) transcriptional signatures of stemness or restrained states in bulk or single-cell RNA sequencing datasets; and (iv) circulating CD4 TSCM cell frequencies, which correlate with disease outcomes in autoimmune contexts (170–172) and may extend to cancer.

### 6.5 Implications for immunosuppressive therapies

The stem-like CD4 T cell framework not only suggests strategies to enhance antitumor immunity but also provides a unifying explanation for the limited durability of conventional immunosuppressive therapies. The recurrent pattern of disease control during treatment followed by relapse upon withdrawal in transplantation and autoimmunity may, in part, reflect the persistence of a stem-like T cell reservoir.

Calcineurin inhibitors (CNIs), including tacrolimus and cyclosporine, suppress effector differentiation by inhibiting NFAT signaling and reducing TOX and PD-1 expression, thereby limiting progression to terminal exhaustion (173). In allogeneic hematopoietic stem cell transplantation models, CNIs maintain alloreactive CD4 T cells in a TCF1^+^ central memory–like state with self-renewal and proliferative capacity but minimal effector function (173). Consequently, although acute graft-versus-host disease is controlled, a persistent reservoir of pathogenic T cells is preserved; upon drug withdrawal, these cells rapidly differentiate into effectors and drive relapse (173).

mTOR inhibitors, such as rapamycin, exert related effects through distinct mechanisms. mTOR signaling is required for the metabolic transition from stem-like progenitors to effector states; its inhibition maintains TCF1 expression and stabilizes memory-like populations (16, 174). In infection models, rapamycin restricts terminal TH1 differentiation of CD4 T cells, thereby preserving the stem-like progenitor pool (97). Together, these findings indicate that immunosuppressive therapies control acute responses by limiting effector differentiation while preserving stem-like T cell populations capable of reactivation.

This framework has dual implications for tumor immunotherapy. Moderate mTOR inhibition may preserve long-term immune potential by maintaining the stem-like CD4 T cell reservoir, but concurrently limits metabolic reprogramming required for effector differentiation. Accordingly, the use and timing of immunosuppressive and immunostimulatory interventions should be guided by the dynamic balance between stem-like and effector T cell states, rather than by global enhancement or suppression of immunity. Such calibration is necessary to avoid sustaining ineffective or pathogenic reservoirs while achieving adequate short-term effector responses.

## 7 Remaining controversies and challenges

The stem-like CD4 framework is conceptually useful, but four controversies currently limit mechanistic and clinical interpretation: cross-tumor generalizability, the definition of the progenitor state, the relative importance of cell-intrinsic versus environmental regulation, and the lack of intervention studies that isolate this population in patients.

First, it is unknown whether comparable stem-like CD4 states exist across all tumor types. Most functional data come from selected mouse models and immunologically active renal, bladder, or prostate tumors (18), while pan-cancer atlases provide mainly transcriptional rather than functional support (19). Studies in cold tumors, metastatic sites, and treatment-naïve versus treated lesions should use harmonized marker panels and functional assays. Negative results will be as informative as positive findings because they will define the tumors in which this framework is applicable.

Second, the intracellular and extracellular determinants of fate must be separated experimentally. TCF1, BCL6, TOX, IRF4, and metabolic programs may alter competence within the cell, whereas Treg cells, dendritic cells, cytokines, antigen strength, and nutrient availability determine which competence is expressed. Factorial perturbations that combine cell-intrinsic editing with controlled environmental changes are needed to identify interactions rather than single-factor associations.

Third, current populations may contain true multipotent cells together with lineage-biased progenitors. Bulk transfer and clonotype overlap support developmental relatedness but cannot prove that an individual cell self-renews and generates multiple fates. Barcoded single-cell transfer, inducible lineage tracing, and longitudinal multi-omics should test this directly. In human tumors, paired TCR tracking across TDLNs, tumors, blood, and serial samples will be essential because invasive lineage tracing is not feasible.

Fourth, the TDLN-centered CD4–CD8 axis remains a working model. Critical experiments include CD4-specific IFN-γ deletion, CD8-specific IFN-γ-receptor perturbation, dendritic-cell licensing controls, and spatial measurement of cognate interactions in intact TDLNs. These studies should determine whether IFN-γ acts directly on CD8 progenitors, indirectly through antigen-presenting cells, or as part of a broader inflammatory transition.

Therapeutic translation must also address evidence specificity and safety. Checkpoint combinations, Treg-directed antibodies, cytokine delivery, metabolic modulation, and CAR TSCM products are clinically relevant, but most have not been shown to work by targeting endogenous stem-like CD4 T cells. Prospective trials should prespecify pharmacodynamic endpoints for the stem-like, TH1, Tfh, cytotoxic, and Treg compartments. Because fate release may also activate autoreactive clones, tumor-local delivery and reversible interventions may offer safer tests of causality.

A high-priority research program would therefore combine harmonized phenotyping, spatial analysis, clonal lineage reconstruction, metabolic-flux measurements, and perturbation studies across multiple tumor types. Success should be judged by causal control of fate, durable antitumor benefit, preservation of the progenitor reservoir, and absence of clinically meaningful autoimmunity—not by changes in TCF1 frequency alone.

## 8 Conclusion

The identification of TCF1^+^PD-1^+^ lin^-^ CD4 populations provides an emerging framework for CD4 T-cell biology in tumors. The framework proposes a candidate progenitor-like compartment whose population-level persistence and context-dependent output may help sustain immune responses. However, durable self-renewal and multipotent output have not yet been demonstrated in the same individual tumor-associated CD4 T cell, and the most detailed direct tumor evidence remains concentrated in selected models. These cells should therefore be viewed as a promising upstream candidate population rather than an established central regulatory node shared by all tumors.

In tumors, fate decisions are associated with a balance between TCF1–BCL6–BACH2 programs that favor progenitor maintenance and IRF4–LDHA programs that enable effector differentiation. Treg cells, myeloid and stromal populations, TGF-β, cytokine availability, and metabolic stress further shape this balance. A proposed working model links TH1 differentiation in TDLNs to downstream CD8 effector generation, but the direct target of CD4-derived IFN-γ and the contribution of dendritic-cell licensing remain unresolved. Enforced TBET expression demonstrates that fate can be experimentally redirected, although evidence from adoptive systems should not be generalized to all endogenous tumor-specific cells.

Therapeutically, combinations that activate the CD8 progenitor pool while releasing or redirecting CD4 differentiation may improve antitumor responses. Treg depletion, cytokine delivery, metabolic modulation, and stemness-preserving adoptive products provide testable routes, but most lack direct clinical evidence that the benefit is mediated by endogenous stem-like CD4 T cells. The persistence of CD4 CAR-T clones and the superior expansion of CAR TSCM products support feasibility of durable less-differentiated T cell states, while leaving the responsible CD4 phenotype to be defined.

However, important limitations remain. The mechanisms underlying the restrained state, the molecular basis of the CD4–CD8 cooperative axis, and the definition of stemness require rigorous validation. Most fate-redirection strategies have been demonstrated in transplantation or adoptive transfer models, and their relevance to endogenous cells and across tumor types is unclear. Furthermore, interventions that promote effector differentiation may also activate autoreactive clones, necessitating careful preclinical evaluation of safety.

Two developments are likely to drive near-term advances: direct validation of the spatial organization of the CD4–CD8 cooperative axis using spatial transcriptomics, and prospective clinical evaluation of stem-like CD4 T cell differentiation states as predictive biomarkers. As CCR8-directed Treg-targeting agents and TIGIT-blockade strategies progress through clinical development, combination strategies aimed at redirecting stem-like CD4 T cell fate may transition from mechanistic models to clinically actionable approaches.
