# Correction: Stem-like CD4 T cells in tumors: an emerging framework for antitumor immunity

**DOI:** 10.3389/fimmu.2026.1984763

**Published:** 2026-09-14

**Authors:** Jiaxiang Xu, Yunchao Yang, Jun Zhang, Yue Zhang

**Affiliations:** 1 Affiliated Hospital of Shandong University of Traditional Chinese Medicine, Jinan, China; 2 Shandong College of Traditional Chinese Medicine, Yantai, Shandong, China; 3 Shandong University of Traditional Chinese Medicine, Jinan, China

**Keywords:** cancer immunotherapy, immune checkpoint blockade, stem-like CD4 T cells, T cell differentiation, T cell stemness, TCF1, tumor immunity, tumor microenvironment

Affiliation “Affiliated Hospital of Shandong University of Traditional Chinese Medicine, Jinan, China” was erroneously given as “Department of Emergency Surgery, Affiliated Hospital of Shandong University of Traditional Chinese Medicine, Jinan, China”.

The original version of this article has been updated.

